# Time for a Drink? A Mathematical Model of Non-human Primate Alcohol Consumption

**DOI:** 10.3389/fams.2019.00006

**Published:** 2019-02-22

**Authors:** Sharon Moore, Ami Radunskaya, Elizabeth Zollinger, Kathleen A. Grant, Steven Gonzales, Erich J. Baker

**Affiliations:** 1 Department of Computer Science, Bioinformatics, Baylor University, Waco, TX, United States; 2 Department of Mathematics, Pomona College, Claremont, CA, United States; 3 Department of Mathematics and Computer Science, St. Joseph’s College, Brooklyn, NY, United States; 4 Oregon Health & Science University, Portland, OR, United States

**Keywords:** mathematical model, drinking classification, Markov process, stochastic, model fitting, alcohol consumption

## Abstract

We simulate a non-human primate’s alcohol drinking pattern in order to better understand temporal patterning of alcoholic drinks that can lead to the excessive intakes associated with alcohol use disorder. A stochastic mathematical model of alcohol consumption pattern is developed, where model parameters are calibrated to an individual monkey’s drinking history. The model predicts a time series that simulates a monkey’s alcohol intake in time, and we analyze this drinking pattern to understand the variations in day and night drinking, the lengths of drinks (intake in 5 or more consecutive secs), and lengths of bouts (1 or more drinks per 5 min occasion). This time series can predict a lifetime categorical drinking level (light, binge, heavy, or very heavy), thus correlating an individual monkey’s parameters with distinct long term drinking classifications.

## 1. INTRODUCTION

Animal models in alcohol research incorporate wide experimental variation, including genetic manipulation, longitudinal observation, and heredity studies [1] to elucidate determinants of consumption as well as potential therapeutics. The Non-Human Primate (NHP) oral ethanol self-administration model has the benefit of more closely approximating human physiological, neuroanatomical and social influences on ethanol consumption then rodents, for example. Our NHP model has been a powerful tool for recreating experimental conditions reflective of Alcohol Use Disorder (AUD) in humans [2, 3]. This model has been successfully employed to measure the driving forces and effects of drinking under numerous conditions and for a variety of biological processes, including predicting future drinkers based on induction drinking [4], classification of drinkers based on age of intoxication [5], epigenetic determinants [6], and effects on bone [7], hormones [8], and neuro-anatomical [9] and brain transcriptome features [10], among others. The strengths of this approach relies in its ability to track robust longitudinal data points and reproducibility across cohort designs that maintain core procedural integrity.

However, many normative human behaviors cannot be faithfully recapitulated in any model organism. The trajectories from a person’s first alcoholic drink to a diagnosis of AUD is an active area of research for diagnostic and prevention purposes [11, 12]. To date, the vast majority of these studies have been retrospective analyses of imprecise definitions of a “drink”. This is changing with advanced monitoring devices; however, studies of human naturalistic drinking still has severe limitations on controlling organismal and environmental variables. Although the use of NHPs can control and track many of the drinking patterns that impede human studies of AUD, including measurements in real time, gender, nutritional and medical status, etc., accurate measures of voluntary alcohol intakes that capture the excessiveness of chronic alcohol drinking associated with AUD also remain difficult aspects of animal models. Likewise, effective treatments for AUD could improve if drinking patterns provided early identification of successful as well as unsuccessful attempts to control intake. Thus, a well-defined mathematical model may represent an important approach to developing an instrument that captures stable, longterm drinking trends, predicts harmful drinking levels, and explores intervention strategies.

In this paper we present a mathematical model that describes the drinking behavior of individual monkeys using data collected as part of the Monkey Alcohol Tissue Research Resource (MATRR) [13], a tissue repository and analytics resource for NHP drinking experiments. The goal of the model is to predict drinking behavior based on organismal features such as those measured in the experiments described above. Previous mathematical models of human alcoholic drinking include compartment models, where each compartment represents a subset of the population. For example, in Benedict [14] alcoholism is modeled as an infectious disease, where the population is divided into Susceptible, Infected (addicted to alcohol), and Recovered classes. Differential equations are used to describe the evolution of alcoholism in the population, mimicking the spread of a contagious disease, allowing the authors to describe critical parameter values, such as the reproduction number, familiar from the traditional **SIR** models used to predict the onset of epidemics [15]. A modification of this model is presented in Bhunu [16], where a fourth compartment consisting of drinkers who are not addicted is added. These deterministic models focus on determining strategies to control drinking behaviors through, for example, limiting the consumption of alcohol in the susceptible population. A more complex compartmental model is described in Ackleh et al. [17] where the population is further subdivided into abstainers, light drinkers, moderate drinkers, problem drinkers, and binge drinkers. Movements between groups are affected b y an individual monkey’s characteristics (“risk”), social interactions, such as individual monkeys from two different groups meeting each other and affecting each other’s behavior, and social pressure (seeing other people in their community engage in e.g. binge drinking). After fitting model parameters to data from college campuses, the model is used to explore the effect of college policies on the prevalence of drinking.

Compartment models describe the behavior of a population using averages over a typically small number of subgroups. Agent-based models, such as the one described in Gorman et al. [18], can incorporate more complex interactions between individual monkeys. Another way to capture variation between individual monkeys is to describe their behavior using a probability distribution. A non-parametric stochastic model using a Hidden Markov model is described in Maruotti and Rocci [19] . In this model, individual monkeys are categorized as being in one of three states: non-drinking, moderate drinking, and heavy drinking. A finite-mixture model is used to characterize individual monkeys according to a fixed set of characteristics.

Other models attempt to predict addictive behavior, for both alcohol and tobacco consumption, based on an individual monkey’s features [20, 21] . Our ultimate modeling quest is to use an individual monkey’s features to inform a parametric stochastic model of alcohol consumption. The model that we propose is outlined in section 3. It describes drinking behavior over time as an embedded Markov Chain, with transition probabilities and rates assumed to be functions of physiological features of the individual monkeys. In this paper we describe the model itself, comparing simulations to experimental data.

## 2. METHODS

### 2.1. Animals

#### 2.1.1. Monkeys

Models were constructed from twenty-three rhesus monkeys (*Macaca mulatta*) collected as part of larger animal cohort studies from the Oregon National Primate Research Center (ONPRC). This study includes cohorts designated “4,” “5,” and “7b,” Table 1, a subset previously reported in classification [3] and prediction studies [4].

All animals were born in pedigreed populations at the ONPRC (Oregon National Primate Research Center) and remained in social groups until a minimum of three months prior to the onset of oral ethanol self-administration. At that time they were transitioned to individual cages according to established protocols [5].

#### 2.1.2. Ethics

The alcohol self-administration protocol was reviewed and approved by the Oregon Health & Science University Animal Care and Use Committee and was in accordance with the guidelines for the care and use of laboratory animals (Guide for the Care and Use of Laboratory Animals; Institute for Laboratory Animal Research, National Research Council, National Academy Press, 2011).

#### 2.1.3. Ethanol Self-Administration Protocol

Adapted from the rodent literature, Schedule Induced Polydipsia (SIP) in primates has been an effective protocol to establish voluntary ethanol consumption (i.e., self-administration) through the use of interval schedules of food delivery [2]. Briefly, as described in Grant et al. [2], animals were induced to drink water and ethanol (4% w/v in water) after training to operate a drinking panel affixed within their housing cage. Banana-flavored food pellets (1 g) were delivered every 300 seconds (for a fixed time of 300s) until the intended volume of fluid was consumed. After water only SIP, monkeys were induced to increasingly large volumes of ethanol in 30 day increments, starting at 0.5 g/kg/day (52-74 ml), and progressing to 1.0 g/kg/day (110-147 ml), then 1.5 g/kg/day (170-223 ml). Following SIP induction, the monkeys were allowed a choice of water or ethanol, each fluid concurrently available, for 22 hrs/day in a phase of the protocol known as “open-access.” Food was available in the form of 3 meals of similar size (number of 1 g food pellets), at approximately 2 hr intervals. Each daily session of 22 hrs began in the late morning and ended in the next calendar day’s early morning. The lighting schedule in the room was 11 hrs light, 13 hrs dark; the lights went off 7 hrs after the start of the open-access session.

### 2.2. Data Collection

#### 2.2.1. Physical Data Collection

Drinking panels with custom acquisition interfaces were used to capture self-administration data and food intake patterns [2, 22]. Each animal has a choice of 4% ethanol by w/v diluted by water or water, each attached to a scale with an accuracy of ±0.1 gram, where each gram is equivalent to one ml (O’Haus Corporation, Parsippany, NJ). Scales are continuously measured by custom Labview (National Instruments Corporation, Austin, TX) hardware and software; resulting data is cleansed for mechanical failures or outliers created from displaced bottles or malfunctioning dispensers.

#### 2.2.2. Drinking Categories

Each animal was classified into one of four well-defined drinking categories (LD, BD, HD, or VHD) based on percentage of open-access days of thresholded intake volumes, modified slightly from previously described methodology [3]. VHD animals have average daily intakes > 3 g/kg with more than 10% of days exceeding 4 g/kg; HD is categorized by ethanol intakes of > 3 g/kg per day for more than 20% of open-access days; BD exhibit > 2 g/kg ethanol intake for more than 55% of open-access days, with a minimum of at least one Blood Ethanol Concentration (BEC) event of > 80 mg/dl. Remaining animals are categorized as LD. For this study, VHD and HD animals are routinely condensed into a single *heavy* drinking category. BD and LD are likewise collapsed into a single *non-heavy* category. This increases statistical power of the collapsed category for model testing and circumvents the need for including BEC as a discriminating factor as BEC data availability is currently not included in the predictive modeling.

#### 2.2.3. Drinks and Bouts

Intake of ethanol and water are discretized events called *drinks*. Drinks have a minimum effective resolution of 0.1 second and 0.1 mg of fluid, and continue as a single episode until the completion of an event. Less than 5 seconds between events is considered a single drink. A *bout* is defined as the volume of ethanol consumed without a 5-minute lapse between *drinks*, as defined by a previous principle component analysis of drinking patterns [2].

#### 2.2.4. MATRR

The Monkey Alcohol Tissue Research Resource [13] can be found at http://www.matrr.com. It aims to maximize the use of NHP resources by limiting experimental redundancy, provide a repository of uniformly derived tissues and data imperative for long term alcohol research, and enhance cross-discipline integration. Data include drinking behavior on a per-second basis, blood protein and hormone information, molecular and genome data, imaging, and animal meta data among others. Previous research has used publicly available data to classify stable drinking categories in NHP [3] and enable the prediction of future drinkers based on early drinking behaviors [4].

#### 2.2.5. MATRR Computing Analytics

All data was collated and analyzed on the MATRR computing servers, twin computers, each running 4 Intel Xeon E5620 processors (Intel Corporation, Santa Clara, CA) at 2.4 GHz, having 4 cores per processor (16 cores per server), with 47 GB of memory. Statistical analysis and data processing was performed using Python and R.

## 3. DEVELOPMENT OF THE MATHEMATICAL MODEL

The mathematical model of the drinking features that we develop in this section is a stochastic process that predicts second by second drinking of an individual monkey. At each time point, a monkey is in one of four states: not drinking (state 1), low (state 2), medium (state 3), or high (state 4) drinking. Transitions between each state, as well as the time spent in a given state, are described by random variables whose distributions can be calibrated to each individual monkey. Once a monkey’s drinking is simulated, the average daily alcohol consumption is calculated to classify the monkey as a *non-heavy* or *heavy* drinker as described in section 2.2.2. These two categories are further refined. The *non-heavy* category is sub-divided into light drinkers (LD) and binge drinkers (BD), while the heavy category is made up of heavy drinkers (HD), and very heavy drinkers (VHD). These sub-categories are calculated from the average daily ethanol intake (ADEI) as described in section 2.2.2, with the exception of the binge category: since the current mathematical model does not predict BEC, a simulation is classified as a “binge drinker” if it has not been classified as HD or VHD, and the ADEI is greater than 2 g/kg on more than 55% of the simulated days.

### 3.1. Markov Model

**FIGURE 1 |:**
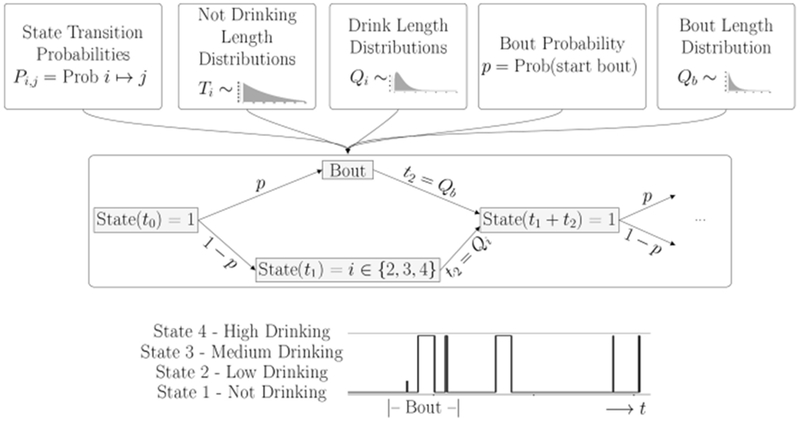
Overview of Model: Once all parameters for the probability distributions are estimated from the data, the simulation is run for an individual monkey. The probabilities determine if the monkey is in a bout or not, and the drinking state. The distributions determine how long the monkey stays in each state. The bottom panel shows a short piece of a time series generated by a typical simulation. A *Bout* is defined as a sequence of drinks where the intervals between drinks is less than 300 s. In this simulation, we see a bout consisting of one drink with a low drinking rate, followed by two drinks at a high rate, and then three single drinks of varying lengths at a high drinking rate.

We describe the drinking process as a continuous time Markov process on the four states: not-drinking, drinking at a low, medium or high rate. The transitions between these states are given by a set of probabilities that depend only on the current state, and not on the past history. In addition, a stochastic *rate* process determines the time spent in each state. In this model, there are two sets of rate processes, depending on whether a monkey enters a *bout* or not. A bout is a sequence of drinks such that the time between drinks is less than 300 seconds. If the time between drinks is more than 300 seconds, the bout has ended, and the decision to enter a new bout or not is made according to a fixed probability, *p*. The lengths of the bouts is described by the distribution, *Q_b_*(*t*). A diagram illustrating the model flow is shown in Figure 1.

The Markov transition matrix describing the probabilities of moving from state *i* to to state *j* with Prob(*i* ↦ *j*) = *P*(*i*,*j*) is:

$$P=\left[\begin{array}{cccc} 0 & P_{nl} & P_{nm} & P_{nh}\\ 1 & 0 & 0 & 0\\ 1 & 0 & 0 & 0\\ 1 & 0 & 0 & 0 \end{array}\right]$$

**FIGURE 2 |:**
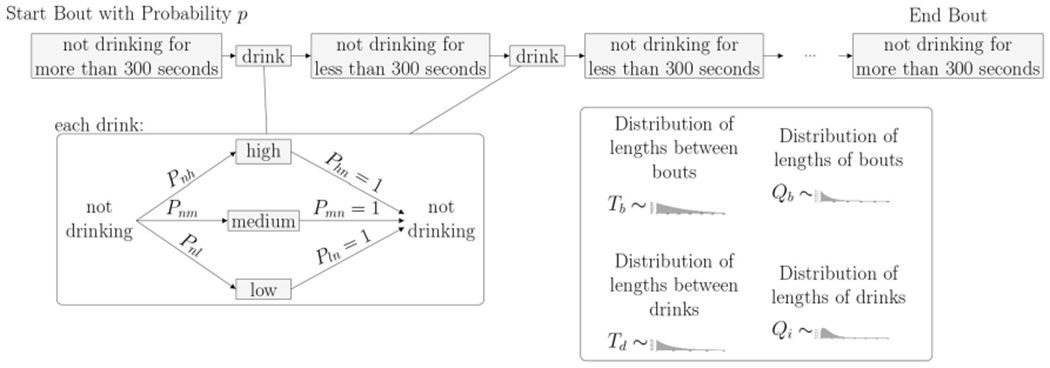
Bouts and Drinks: The length of time between drinks determines if a monkey is in a bout or not. When not in a bout, a monkey takes an individual drink. When in a bout, a monkey takes several drinks in a row with short times in between. Regardless of whether or not the monkey is in a bout, a Markov Process dictates the level of each drink where state 1 is not drinking, state 2 is low, state 3 is medium, and state 4 is high.

As shown in the transition matrix, we assume that, after every drinking state, the monkey enters the non-drinking state with probability 1. Probabilities of transitioning to one of the drinking states are given in the top row of the matrix. We note that these probabilities, *P_nl_*, *P_nm_* and *P_nh_*, as well as the distributions giving the time spent in each state, can be estimated from the drinks taken by each individual monkey. In order to implement the model, we will need to estimate these transition probabilities, as well as the time spent in each state. Based on the experimental data, we choose to use five different probability distributions for the times between states. The distributions *T_d_*(*t*) and *T_b_*(*t*) describe the time spent between drinks in a bout or between bouts, respectively. The distributions *Q_i_*(*t*) describe the time spent in drinking state *i*, where *i* = 2 (low drinking rate), 3 (medium drinking rate) and 4 (high drinking rate) (see Figure 2).

### 3.2. Implementation

**FIGURE 3 |:**
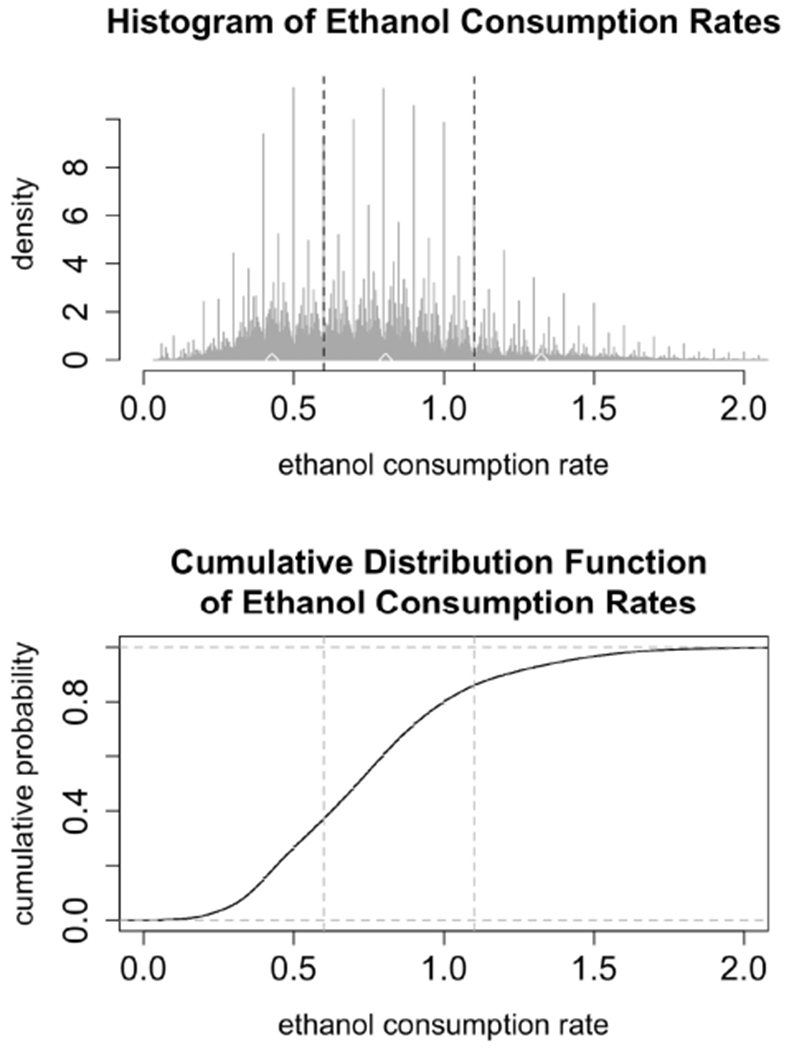
**(Top)** The histogram shows 22 h of drinking per day for a year for 31 monkeys, not including 1 s drinks at a rate less than or equal to 0.2 mL/s. The vertical black dashed lines denote breaks between the low, medium, and high categories: low drinking is (0, 0.6), medium is [0.6, 1.1], and high is above 1.1. The upper and lower cut-off for each bin were derived from the data by finding local minima in the (smoothed) histogram. The median values are marked by white diamonds: median low is 0.383, median medium is 0.798, and median high is 1.376. In the simulations, low, medium and high rates are set at these median levels. **(Bottom)** The cumulative distribution function of ethanol consumption rates plot presents another view of the same ethanol consumption rates data as in the upper panel. The vertical dashed lines represent the breaks between low, medium, and high drinking rates.

To estimate the transition probabilities, *P_nj_* and to estimate the parameters of the distributions describing the times spent in each state, we start by examining the data for 31 monkeys, showing 22 hours of drinking per day for a year. We filter out drinks that are 1 second long and contain 0.2 mL of alcohol consumption. These drinks represent the lowest possible value that can be recorded and could be an indicator of slight disturbances in the balances, such as air bubbles in the tubing. As seen in the histogram in Figure 3 we classify the rates into three bins representing low, medium, and high drinking. The upper and lower cut-offs for each bin were derived from the data by finding local minima in the (smoothed) histogram. Any drink with a rate between 0 and 0.6 is considered a *low* drink; any drink between (and including) 0.6 and 1.1 is considered a *medium* drink; drinks with a rate above 1.1 are considered *high* drinks. In the simulations, we use the median values of each of the bins. We used the binned data to estimate the transition probabilities (*P_nl_*, *P_nm_*, and *P_nh_*) as the fraction of drinks that were in each category, as well as the probability of entering a bout (*p*). Note that we are assuming that the probability of entering a given drinking state (low, medium or high) is independent of the past drinking history.

For each drinking and non-drinking state we extract from the experimental data the corresponding drink lengths and breaks between drinks. A first exploration of the distributions of these data showed that the time spent in each drinking state was well-described by a Weibull distribution. This makes sense, since a Weibull distribution models a process for which the average number of events per unit time (the rate) is a power of the amount elapsed. The value of this power is called the *shape* of the distribution. Thus, if the shape parameter is 1, the Weibull is the same as an exponential distribution. If the shape parameter is greater than one, it means that the rate of events increases with time; in our context, it means that a monkey who is drinking is more likely to stop drinking in the next second as the drink length increases. As shown in Figure 1, the time between drinks, or time in state 1, is described by two random variables: *T_d_*, the time between single drinks, defined as times less than 300 seconds, and the *T_b_*, the time between bouts, defined as non-drinking times greater than 300 seconds.

We fit the parameters of the corresponding distribution to the data using a best-fit function in R. Table 2 shows a summary of the shape (*k_i_*) and rate (λ_*i*_) parameters for each of the drinking states: 1 (not drinking), 2 (low drinking rate), 3 (medium drinking rate), and 4 (high drinking rate). We note that *Q_b_* (length of bouts), *T_b_* and *T_d_* had shape parameters equal to 1, i.e., they are well-described by an exponential distribution.

**FIGURE 4 |:**
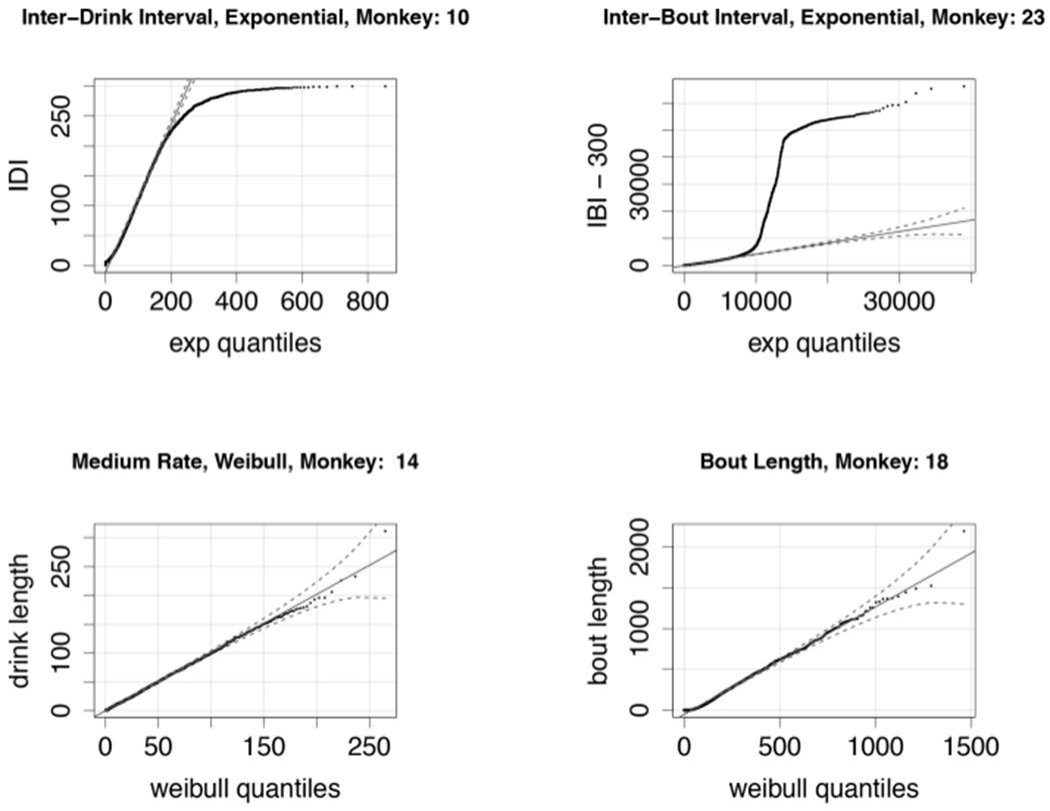
**(Top Left)**: A quantile-quantile (q-q) plot of the distribution of experimentally derived inter-drink intervals against the best-fit exponential distribution; note that inter-drink intervals are always less than 300 s, explaining the deviation from the exponential quantiles at the high end. This particular monkey (ID # 10) is classified as a *heavy* drinker. **(Top Right)** A q-q plot of the distribution of experimentally derived inter-bout intervals against the best-fit exponential distribution. Inter-bout intervals are all greater than 300 s, so we subtract 300 from all data points before fitting (these shifted data points are shown here). The sharp vertical deviation of the plot from the fitted quantile plot from ca. 9000 s (2.5 h) and 45,000 s (12 h) suggests that this monkey (ID # 23) took very few drinks during the night-time. This is consistent with the monkey’s classification as a *light* drinker. **(Bottom Left)** A q-q plot of drink lengths of a heavy drinker (ID # 14), when drinking at medium rate. The distribution fits a Weibull very well for all drink lengths. **(Bottom Right)** The bout lengths for a light drinker (ID # 18) also fit a Weibull distribution quite well for a range of bout lengths.

Figure 4 shows comparisons of the experimentally derived values to the best-fit distributions. We note that the fits are quite good for a range of drinking behaviors, with the exception of the distribution of the length of times between bouts, i.e., inter-drink lengths that are greater than 300 s. The fit is good for times up to 10,000 s, or just under 3 h, after which the data shows a greater frequency of longer times than an exponential model would predict. This is consistent with our observation that certain individual monkeys, in particular light drinkers, do not drink during the night time. We discuss model refinements that might better reflect this variability in drinking behavior in section 5.

## 4. RESULTS

**FIGURE 5 |:**
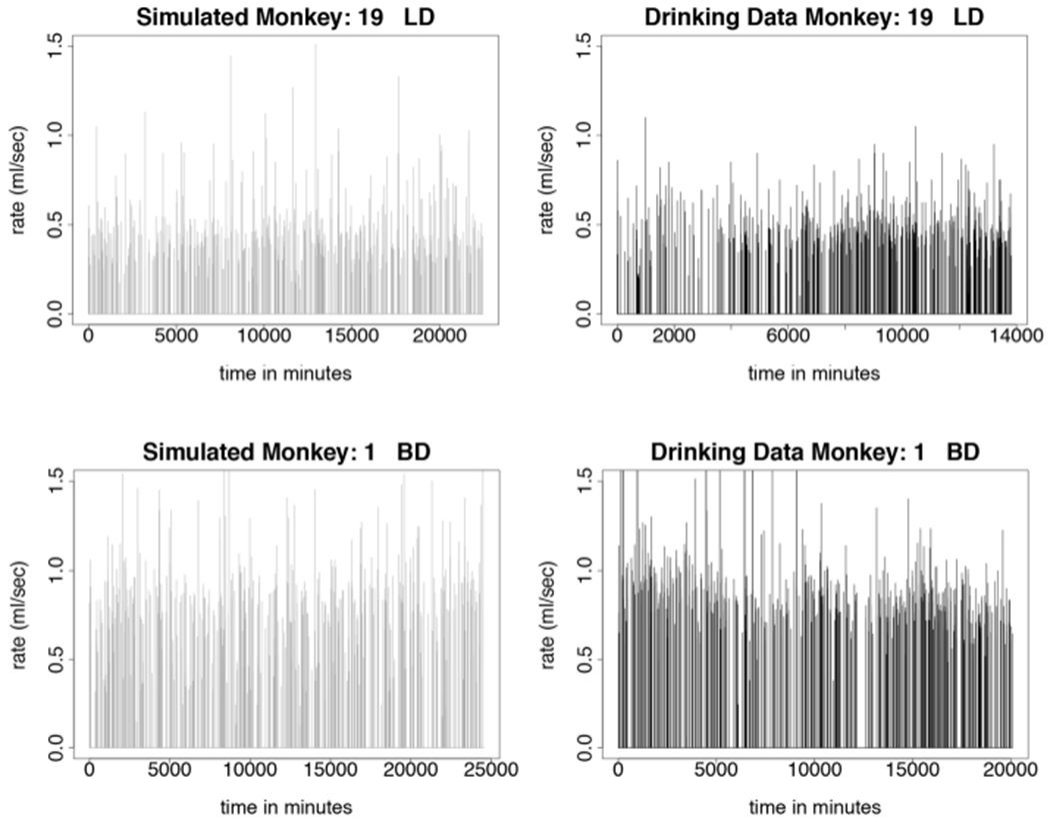
Time series plots comparing simulated drinking data to actual drinking data for light and binge drinkers are shown in this figure. The simulations are qualitatively the same as the data. The light drinkers act like sippers, taking a lot of drinks at a lower rate. Normally distributed noise was added to the simulated values in order to more closely resemble the drinking data. The simulation was run for 180 days, where each day consists only of 7 h of daytime drinking. We use 750 drinks from 12 months of daytime drinking (7 h) in the drinking data figures. **(Upper Left)** Simulated drinking data for a light drinker. **(Upper Right)** Actual drinking data for a light drinker. **(Lower Left)** Simulated drinking data for a binge drinker. **(Lower Right)** Drinking data for a binge drinker.

**FIGURE 6 |:**
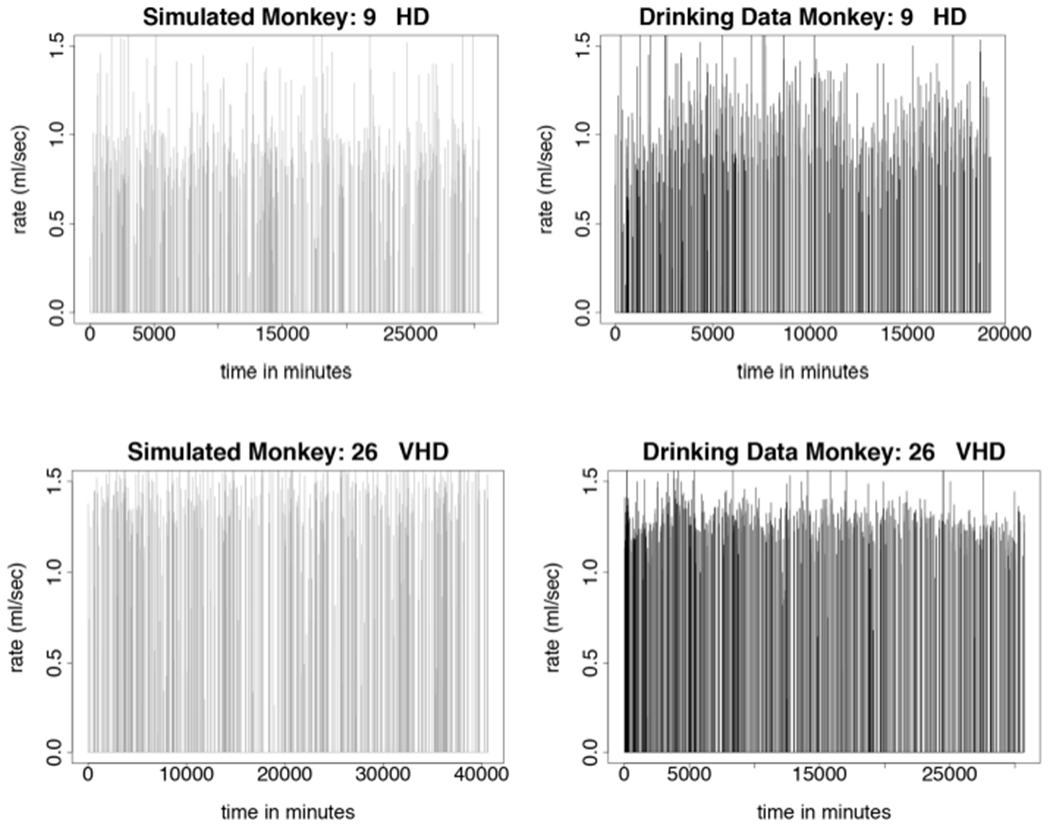
Time series plots comparing simulated drinking data compared to actual drinking data for heavy and very heavy drinkers is shown in this figure. The simulations are qualitatively the same as the data. Instead of sipping like the light drinkers, the heavy drinkers take more gulps as demonstrated by the higher rates of consumption. Normally distributed noise was added to the simulated values in order to more closely resemble the drinking data. The simulation was run for 180 days, where each day consists only of 7 h of daytime drinking. We use 750 drinks from 12 months of daytime drinking (7 h) in the drinking data figures. **(Upper Left)** Simulated drinking data for a heavy drinker. **(Upper Right)** Actual drinking data for a heavy drinker. **(Lower Left)** Simulated drinking data for a very heavy drinker. **(Lower Right)** Drinking data for a very heavy drinker.

We test the model described in section 3 by simulating monkeys from all four drinking categories: light, binge, heavy, and very heavy. Simulations for monkey number 19, a light drinker, monkey number 1, a binge drinker, monkey number 9, a heavy drinker, and monkey number 26, a very heavy drinker, are shown in Figures 5, 6. In our simulation, drinking rates are binned as low (0.383 mL/s), medium (.798 mL/s), and high (1.376 mL/s). These values are obtained from a histogram of all of the drinking data, as shown in Figure 3 and explained in section 3.2. To closer resemble the actual data, we add some noise to the simulated values. We added normally distributed noise with the mean equal to the calculated median value of each bin. For the low bin, the standard deviation is the high limit of the bin minus the median of the bin divided by 2. The other bins are calculated in a similar way. Notice that the light drinkers have drinks mainly around the low drinking rate (0.383 mL/s) and very heavy drinkers have drinks mainly at the high drinking rate (1.376 mL/s). The figures for the drinking data are made from 7 hours of daylight drinking for 12 months of open access. We utilized only 7 hours of daylight drinking each day instead of 22 hours since the monkeys sleep a lot during the night and most of the alcohol consumption is during the day. We run the simulation for 180 days and plot 750 drinks. We chose enough drinks to be able to see several days worth of comparison.

**FIGURE 7 |:**
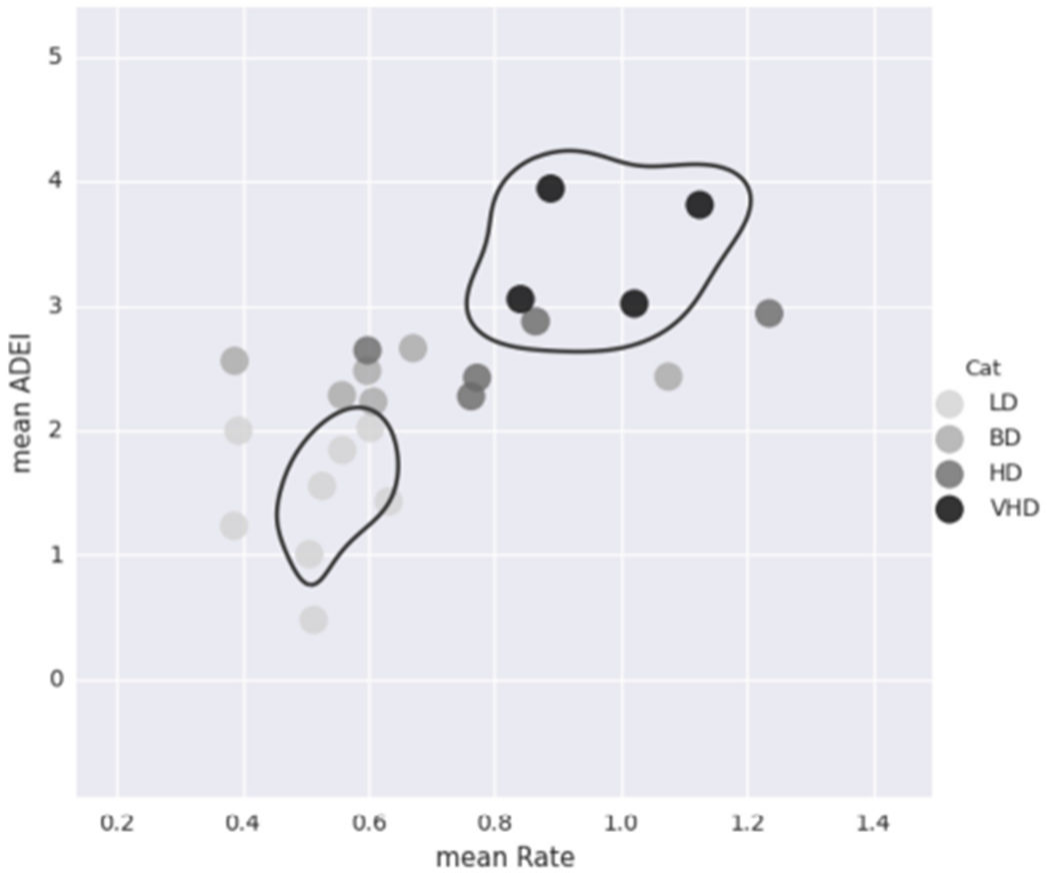
This plot shows the average rate of consumption (mean Rate) in mL/s and the average daily ethanol intake (mean ADEI) in g/kg for all 23 monkeys. Each dot represents one monkey and is color coded by drinking category. Although mean ADEI is very important in determining the monkey’s drinking category, it alone is not enough information. Notice that not all of the heavy drinkers are grouped close together.

For each of the 23 monkeys, we calculate the average rate of ethanol consumption and the average daily ethanol intake. The average daily ethanol intake is calculated in g/kg to account for the different monkey weights. As seen in Figure 7, these values alone are not enough to classify the animals. See section 2.2.2 for a description of how the monkeys are classified into different drinking categories.

Next we looked at the accuracy of the simulation by simulating 23 monkeys, comprised of 8 light drinkers, 6 binge drinkers, 5 heavy drinkers, and 4 very heavy drinkers. We ran the simulation 100 times and calculated the monkey’s drinking category in each simulation as described in section 2.2.2. The results are in Table 3. We record the percentage of times the correct drinking category out of 4 possible categories is identified for 100 simulations. We also collapse the drinking categories into heavy and non-heavy and record the accuracy of identifying the correct category out of just two categories. The light drinkers are correctly identified 100 percent of the time. See section 5 for a more thorough discussion of the results.

## 5. DISCUSSION

Table 3 shows the results from 100 simulations of 23 virtual monkeys. In each simulation, the model parameters were first estimated from the individual monkey’s data, as described in section 3.2. Next, 100 simulations were generated with those parameters, and each of the resulting drinking patterns were classified as LD, BD, HD, or VHD. The table shows the results of these classifications, and also tabulates the fraction of time that the simulations were classified correctly, i.e. if the simulation depicted a monkey in the same class as the original monkey. This gives us two measures of how accurate our model is in predicting drinking class. The sixth column of the table shows the fraction of the simulations that were correctly classified in one of the four groups (“Accuracy 4”), while the seventh column shows the fraction that are correctly classified as either *non-heavy*, (LD or BD) or *heavy*, (HD or VHD). We can see from this table that the simulations of light drinkers are accurately classified in both cases, while the other categories show some inconsistencies. In fact, 100% of the light drinkers are classified correctly 100% of the time and three out of four of the very heavy drinkers are correctly identified 100 % of the time when the drinking categories are collapsed into heavy and non-heavy drinkers. Overall, approximately 80% of the monkeys are classified correctly in at least 95% of the simulations. The binge category has the lowest accuracy and is one of the hardest to classify. The definition of a binge drinker requires the monkey to have at least one binge which is represented by a BEC above 80 mg/dl. Our current model does not include a BEC model, thus for our future work we plan on incorporating a BEC model (see section 6). The inconsistencies in the classifications can be explained by several factors, and suggest (at least) two improvements to our model.

**FIGURE 8 |:**
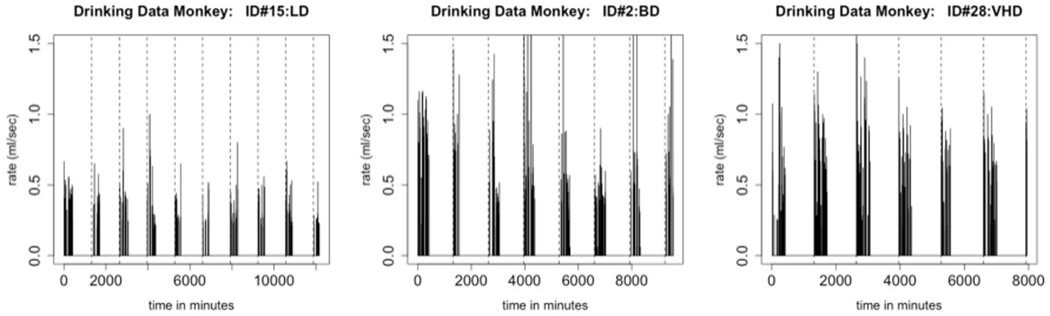
This figure shows an individual monkey’s rate of consumption for 700 drinks, which is several days. The figure on the left is a plot of a light drinker, the middle figure is a plot of a binge drinker, and the figure on the right is a plot of a very heavy drinker. There is a substantial difference between daytime drinking and nighttime drinking. Each dashed line represents the end of a day. For all types of drinkers, there are shorter gaps between drinks during the daytime than at night. Notice the large breaks in drinking at the end of the day, which is when the monkey is sleeping.

One of our model assumptions was that the distributions of times between drinks and of transitions between drinking states are stationary; in particular, they do not depend on the time of day. This assumption is questionable when we examine the data more closely. Consider the time series shown in Figure 8. We note that, in all three types of drinkers, there are longer breaks during the night-time hours than during the early part of the day, giving evidence that the distribution of times between drinks is *not* stationary. We propose that, because the distributions of inter-bout times for light drinkers will have very light tails, this non-stationarity has less of an effect when simulating drinkers in the LD class. In future work, we plan to use two distributions of inter-bout times for each monkey, one that describes day-time drinking, and one that describes night-time drinking.

**FIGURE 9 |:**
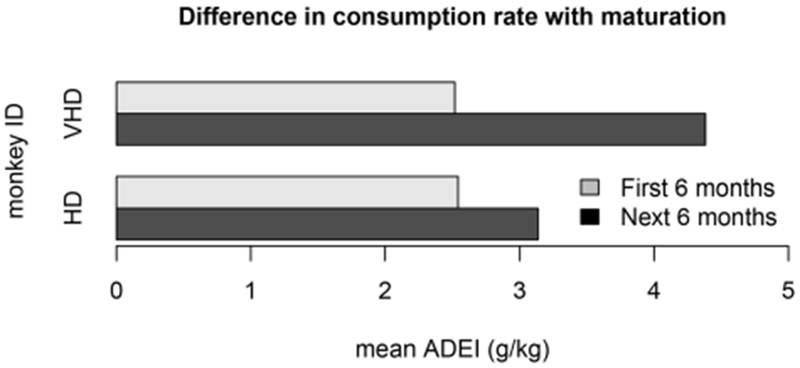
This bar plot shows the difference in ethanol consumption during the first 6 months of open access and the second 6 months of open access for 2 monkeys in cohort 7a. The monkeys matured from adolescence to adulthood during the open access period, which substantially impacted their weight and drinking patterns. The ethanol consumption rate for the first 6 months is different than the second 6 months for all of the monkeys in the cohort. This plot shows the average daily ethanol intake for one very heavy drinker and one heavy drinker.

Another violation of the non-stationarity assumption is the result of drinking behavior that changes as the monkeys age. One cohort of monkeys matured from adolescence to adulthood during the open access period. The mean average daily ethanol intake is shown for two different monkeys from this cohort in Figure 9. We note that the first 6 months of open access had very different ethanol consumption rates than the second 6 months of open access, and the magnitude of these differences may depend on the drinking type of the monkey. Another model refinement that we plan to implement is to estimate different distributions for open access periods at different states of an individual monkey’s development.

In summary, we have developed and calibrated a mathematical model of drinking behavior that produces simulations of individual monkeys that are accurately classified 80% of the time. The accuracy is much better in the class of light drinkers, which suggests two improvements to the model: inter-drink time distributions that depend on the time of day, and distributions that vary with the age of the monkey.

## 6. FUTURE WORK

One of the deficiencies of the current model is that, while it predicts ethanol intake it does not predict blood ethanol concentration (BEC). In the next iteration of the model, we plan to add BEC to the model output by incorporating a pharmakokinetic-pharmakodynamic (PKPD) model of ethanol metabolism and elimination. PKPD models that describe BEC as a function of ethanol intake have been developed for humans [23–26]. The goal of these models has either been to predict BEC from the analysis of alcohol on the breath [27], or to understand social behavior. We will adapt these models to our environment by adjusting the parameter values to reflect the physiological characteristics of non-human primates. The addition of the BEC model to the current drinking model will accomplish two goals. It will allow us to more accurately classify drinkers, since BEC is used to characterize the BD (binge drinker) class, and we will be able to better relate behavior to ethanol intake, since BEC has been more closely correlated to behavior than ADEI.

In addition, we would like to extend our working model for male rhesus monkeys to include female monkeys. Ethanol consumption of female monkeys is impacted by menses, having a strong correlation with progesterone [28]. By including follicular and luteal phase information in our model, we hope to be able to closely approximate the complexity of NHP drinking in female animals.

In addition, both human and non-human primates are social beings, and drinking behavior among humans has been shown to be affected by social circumstances [29]. Social drinking influences are also observed in rodent studies [30], but have not been quantitative beyond anecdotal evidence in NHP populations. By aggregating individual monkey models, we plan to explore the social effects on the drinking behavior of monkeys by modeling a network of monkeys that reflects cage locations and observed interactions.

**FIGURE 10 |:**
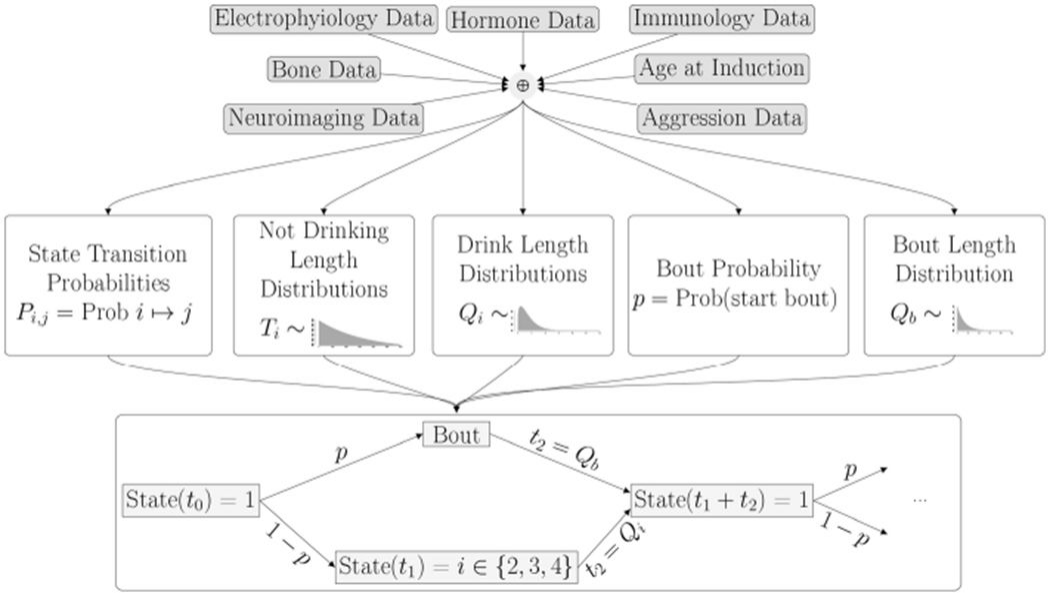
In the next phase of the project, the parameters of the probability distributions will be estimated from an individual monkey’s set of *features* derived from physiological characteristics and measurements.

Now that we have a calibrated model of drinking behavior, we wish to use it to predict drinking behavior from physiological characteristics. In order to do this, we will use data as described in Baker et al. [4] and an unsupervised learning algorithm to estimate the parameters of the model. In other words, we will estimate the parameters that describe the probability distribution functions from the feature data. A schematic of the process, an extension of Figure 1, is given in Figure 10. As a simple example, merely for the purpose of illustration, suppose we have a vector of *n* feature characteristics: *F* = (*a*_1_, *a*_2_, …, *a_n_*) and we want to estimate the three transition probabilities, *P_nl_*, *P_nm_* and *P_nh_* which give the probabilities of transitioning from the non-drinking state to the low, medium and high drinking states, respectively. We might assume that the relationship between the features and these probabilities takes the form:

$$\left[\begin{array}{c} P_{nl}\\ P_{nm}\\ P_{nh} \end{array}\right]=\left[\begin{array}{c} 1-e^{D_{1}\cdot F}\\ 1-e^{D_{2}\cdot F}\\ 1-e^{D_{3}\cdot F} \end{array}\right]$$

where the vectors *D*_1_, *D*_2_ and *D*_3_ will be estimated using an optimization procedure such as a maximum likelihood estimate (MLE).

Our belief is that a general, feature-driven probabilistic model of drinking behavior will help us understand the factors that lead to AUDs. Ultimately, we hope to use the model to independently test the impact of individual characteristics on drinking *in silico*, explore the occurrence and impact of AUDs, and, ultimately, provide a framework to rapidly evaluate pharmacological and behavioral interventions.

## Figures and Tables

**TABLE 1 |:** Three cohorts of Rhesus monkeys were used in this study.

ID	N	Sex	Age (yrs)	Weight (kg)	LD	BD	HD	VHD
**4**	10	M	8.24	9.4	5	4	1	0
**5**	8	M	5.63	8.31	0	1	3	4
**7b**	5	M	5.69	8.02	3	1	1	0

Age and Weight values represent cohort averages. Each cohort is further subdivided into four drinking categories: LD, light drinkers; BD, binge drinkers; HD, heavy drinkers; and VHD, very heavy drinkers.

**TABLE 2 |:** The mean and range of the parameters used in the distributions describing the length of drinks (*Q*_1_, *Q*_2_, *Q*_3_), length of time between drinks (*T_b_*, *T_d_*), and length of bouts (*Q_b_*).

	*Q*_1_		*Q*_2_		*Q*_3_		*Q_b_*		*T_b_*		*T_d_*	
	*k*_1_	λ_1_	*k*_2_	λ_2_	*k*_3_	λ_3_	*k_b_*	λ_*b*_	*k_IBL_*	λ_*IBL*_	*k_IDL_*	λ_*IDL*_
Mean	1.03	16.62	1.14	23.26	1.23	8.63	1.00	141.45	1.00	0.00	1.00	0.01
Min	1.00	1.97	1.00	3.42	1.00	1.28	1.00	47.59	1.00	0.00	1.00	0.01
Max	1.35	40.80	1.49	49.83	1.73	40.94	1.00	341.84	1.00	0.00	1.00	0.03

*We assume a Weibull distribution, and fit the two parameters, k (shape) and* λ *(scale) for each monkey in the data set. We note that the shape parameters for the time between drinks and the length of bouts is one for all monkeys in the data set, indicating that these distributions are well-described by an exponential distribution. Variation in the parameters reflects variations in the drinking patterns of individual monkeys, allowing the model to describe drinkers of all four categories.*

**TABLE 3 |:** One hundred model simulations using parameters estimated from each monkey’s data.

		Counts					

Monkey #	ID	LD	BD	HD	VHD	Accuracy 4	Accuracy 2
15	LD1	100	0	0	0	1	1
16	LD2	100	0	0	0	1	1
17	LD3	100	0	0	0	1	1
18	LD4	100	0	0	0	1	1
19	LD5	100	0	0	0	1	1
23	LD9	100	0	0	0	1	1
24	LD10	100	0	0	0	1	1
25	LD11	100	0	0	0	1	1

1	BD1	0	33	67	0	0.33	0.33
2	BD2	100	0	0	0	0	1
3	BD3	17	83	0	0	0.83	1
4	BD4	0	0	100	0	0	0
5	BD5	0	0	96	4	0	0
7	BD7	100	0	0	0	0	1

8	HD1	0	0	100	0	1	1
9	HD2	0	5	95	0	0.95	0.95
10	HD3	0	0	92	8	0.92	1
11	HD4	17	83	0	0	0	0
14	HD7	35	35	30	0	0.3	0.3

26	VHD1	0	0	0	100	1	1
27	VHD2	0	0	10	90	0.9	1
28	VHD3	0	0	0	100	1	1
29	VHD4	0	100	0	0	0	0

The first column (“ID”) identifies the monkey as a LD, light drinker; BD, binge drinker; HD, heavy drinker; and VHD, very heavy drinker. The second through fifth columns show the number of simulations that were classified as LD, BD, HD, and VHD, according to the definitions given in section 2.2.2. The sixth column shows the fraction of simulations that were classified correctly (“Accuracy 4”) and the final, seventh, column shows the fraction of simulations that are classified correctly when the four categories are collapsed to only two: LD and BD are classified as non-heavy, while HD and VHD are classified as heavy (“Accuracy 2”).

## References

[1] Tabakoff B, Hoffman PL. Animal models in alcohol research. Alcohol Res Health (2000) 24:77–84. 11199281 PMC6713012

[2] Grant KA, Leng X, Green HL, Szeliga KT, Rogers LS, Gonzales SW. Drinking typography established by scheduled induction predicts chronic heavy drinking in a monkey model of ethanol self-administration. Alcoholism (2008) 32:1824–38. doi: 10.1111/j.1530-0277.2008.00765.x 18702645 PMC2847427

[3] Baker EJ, Farro J, Gonzales S, Helms C, Grant KA. Chronic alcohol self-administration in monkeys shows long-term quantity/frequency categorical stability. Alcoholism (2014) 38:2835–43. doi: 10.1111/acer.12547 25421519 PMC4244650

[4] Baker EJ, Walter NAR, Salo A, Rivas Perea P, Moore S, Gonzales S, Identifying future drinkers: behavioral analysis of monkeys initiating drinking to intoxication is predictive of future drinking classification. Alcoholism Clin Exp Res (2017) 41:626–36. doi: 10.1111/acer.13327 PMC5347908 28055132

[5] Helms CM, Rau A, Shaw J, Stull C, Gonzales SW, Grant KA. The effects of age at the onset of drinking to intoxication and chronic ethanol self-administration in male rhesus macaques. Psychopharmacology (2014) 231:1853–61. doi: 10.1007/s00213-013-3417-x 24448900 PMC3969395

[6] Cervera-Juanes R, Wilhelm LJ, Park B, Grant KA, Ferguson B. Genome-wide analysis of the nucleus accumbens identifies DNA methylation signals differentiating low/binge from heavy alcohol drinking. Alcohol (2017) 60:103–13. doi: 10.1016/j.alcohol.2016.11.003 27866807 PMC5420479

[7] Gaddini GW, Grant KA, Woodall A, Stull C, Maddalozzo GF, Zhang B, Twelve months of voluntary heavy alcohol consumption in male rhesus macaques suppresses intracortical bone remodeling. Bone (2015) 71:227–36. 25451322 10.1016/j.bone.2014.10.025 PMC4291183

[8] Helms CM, Park B, Grant KA. Adrenal steroid hormones and ethanol self-administration in male rhesus macaques. Psychopharmacology (2014) 231:3425–36. doi: 10.1016/j.bone.2014.10.025 24781519 PMC4135005

[9] Kroenke CD, Rohlfing T, Park B, Sullivan EV, Pfefferbaum A, Grant KA. Monkeys that voluntarily and chronically drink alcohol damage their brains: a longitudinal MRI study. Neuropsychopharmacology (2013) 39:823–30. doi: 10.1038/npp.2013.259 24077067 PMC3924514

[10] Iancu OD, Colville A, Walter NAR, Darakjian P, Oberbeck DL, Daunais JB, On the relationships in rhesus macaques between chronic ethanol consumption and the brain transcriptome. Addic Biol (2018) 23:196–205. doi: 10.1007/s00213-014-3590-6 PMC5671907 28247455

[11] King AC, Hasin D, O’Connor SJ, McNamara PJ, Cao D. A prospective 5-year re-examination of alcohol response in heavy drinkers progressing in alcohol use disorder. Biol Psychiatry (2016) 79:489–98. doi: 10.1016/j.biopsych.2015.05.007 26117308 PMC4644521

[12] Field M, Jones A, Kersbergen I, Robinson E. Experimental research requires valid and sensitive measures of alcohol intake, and this is a step in the right direction: commentary on leeman and colleagues (2018). Alcoholism (2018) 42:1019–21. doi: 10.1111/acer.13641 29656487

[13] Daunais JB, Davenport AT, Helms CM, Gonzales SW, Hemby SE, Friedman DP, Monkey alcohol tissue research resource: banking tissues for alcohol research. Alcoholism (2014) 38:1973–81. doi: 10.1111/acer.12467 24942558 PMC4370268

[14] Benedict B Modeling alcoholism as a contagious disease: how ‘infected’ drinking buddies spread problem drinking. SIAM News (2007) 40.

[15] Kermack WO, McKendrick AG. A contribution to the mathematical theory of epidemics. Proc R Soc A Math Phys Eng Sci. (1927) 115:700–21. doi: 10.1098/rspa.1927.0118

[16] Bhunu CP. A mathematical analysis of alcoholism. World J Model Simul (2012) 8:124–34.

[17] Ackleh AS, Fitzpatrick BG, Scribner R, Simonsen N, Thibodeaux JJ. Ecosystem modeling of college drinking: parameter estimation and comparing models to data. Math Comp Model. (2009) 50:481–97. doi: 10.1016/j.mcm.2009.03.012 PMC2702788 20161275

[18] Gorman DM, Mezic J, Mezic I, Gruenewald PJ. Agent-based modeling of drinking behavior: a preliminary model and potential applications to theory and practice. Am J Public Health. (2006) 96:2055–60. doi: 10.2105/AJPH.2005.063289 17018835 PMC1751811

[19] Maruotti A, Rocci R. A mixed non-homogeneous hidden Markov model for categorical data, with application to alcohol consumption. Statist Med (2012) 31:871–86. doi: 10.1002/sim.4478 22302505

[20] Kuntsche E, Cooprt ML. Drinking to have fun and to get drunk: motives as predictors of weekend drinking over and above usual drinking habits. Drug Alcohol Depend. (2010) 110:259–62. doi: 10.1016/j.drugalcdep.2010.02.021 20363080

[21] Luo S, Crainiceanu CM, Louis TA, Chatterjee N. Analysis of smoking cessation patterns usinga stochastic mixed-effects model with a latent cured state. J Am Stat Assoc (2008) 103:1002–13. doi: 10.1198/016214507000001030 19305513 PMC2658598

[22] Vivian J, Green H, Young J, Majerksy L, Thomas B, Shively C, Induction and maintenance of ethanol self-administration in cynomolgus monkeys (Macaca fascicularis): long-term characterization of sex and individual differences. Alcoholism (2001) 25:1087–97. doi: 10.1111/j.1530-0277.2001.tb02321.x 11505038

[23] Plawecki MH, Han JJ, Doerschuk PC, Ramchandani VA, O’Connor SJ. Physiologically based pharmacokinetic (PBPK) models for ethanol. IEEE Trans Bio Med Eng (2008) 55:2691–700. doi: 10.1109/TBME.2008.919132 PMC3446827 19126448

[24] Pieters JE, Wedel M, Schaafsma G. Parameter estimation in a three-compartment model for blood alcohol curves. Alcohol Alcoholism (1990) 25:17–24. 2334491

[25] Mumenthaler MS, Taylor JL, Yesavage JA. Ethanol pharmacokinetics in white women: nonlinear model fitting versus zero-order elimination analyses. Alcoholism Clin Exp Res (2000) 24:1353–62. doi: 10.1111/j.1530-0277.2000.tb02103.x 11003200

[26] Umulis DM, Gürmen NM, Singh P, Fogler HS. A physiologically based model for ethanol and acetaldehyde metabolism in human beings. Alcohol (2005) 35:3–12. doi: 10.1016/j.alcohol.2004.11.004 15922132

[27] Pavlic M, Grubwieser P, Libiseller K, Rabl W. Elimination rates of breath alcohol. Forensic Sci Int (2007) 171:16–21. doi: 10.1016/j.forsciint.2006.09.008 17064864

[28] Grant KA, Bennett AJ. Advances in nonhuman primate alcohol abuse and alcoholism research. Pharmacol Therapeut (2003) 100:235–55. doi: 10.1016/j.pharmthera.2003.08.004 14652112

[29] Steele CM, Southwick L. Alcohol and social behavior: I. The psychology of drunken excess. J Person Soc Psychol (1985) 48:18–34. doi: 10.1037/0022-3514.48.1.18 3981386

[30] Varlinskaya EI, Spear LP. Acute effects of ethanol on social behavior of adolescent and adult rats: role of familiarity of the test situation. Alcoholism (2002) 26:1502–11. doi: 10.1111/j.1530-0277.2002.tb02449.x 12394283

